# Insights into MLH1 Methylation in Endometrial Adenocarcinoma through Pyrosequencing Analysis: A Retrospective Observational Study

**DOI:** 10.3390/cancers16112119

**Published:** 2024-06-01

**Authors:** Fábio França Vieira e Silva, Andrea Ballini, Vito Carlo Alberto Caponio, Mario Pérez-Sayáns, Marina Gándara Cortés, Laura Isabel Rojo-Álvarez, Abel García-García, José Manuel Suaréz-Peñaranda, Marina Di Domenico, María Elena Padín-Iruegas

**Affiliations:** 1 Department of Medicine and Dentistry, University of Santiago de Compostela, San Francisco Street, s/n, 15782 Santiago de Compostela, Spain; fabio.francavieiraesilva@unicampania.it (F.F.V.e.S.); mario.perez@usc.es (M.P.-S.); abel.garcia@usc.es (A.G.-G.); jm.suarez.penaranda@usc.es (J.M.S.-P.); 2 Health Research Institute of Santiago de Compostela (FIDIS), Santiago de Compostela University Clinical Hospital, University of Santiago de Compostela, Choupana Street, s/n, 15706 Santiago de Compostela, Spain; marina.gandara.cortes@usc.es (M.G.C.); laurarojoalvarez@gmail.com (L.I.R.-Á.); mepadin@uvigo.gal (M.E.P.-I.); 3 Department of Precision Medicine, University of Campania Luigi Vanvitelli, Via L. De Crecchio, 7, 80138 Naples, Italy; marina.didomenico@unicampania.it; 4 Department of Clinical and Experimental Medicine, University of Foggia, Via Rovelli, 48, 71122, Foggia, Italy; vitocarlo.caponio@unifg.it; 5 Human Anatomy and Embriology Area, Departament of Funcional Biology and Health Sciences, University of Vigo, Lagoas-Marcosende, s/n, 36310 Vigo, Spain

**Keywords:** endometrial adenocarcinoma, pyrosequencing methylation analysis, MLH1

## Abstract

**Simple Summary:**

MLH1 methylation status was accessed by a Pyrosequencing (PSQ) assay, and the results point to the efficacy of PSQ for evaluating MLH1 methylation. While unmethylation appears to be associated with a higher relapse rate, the survival rate does not seem to be influenced by methylation. Quantitative percentages suggest that elevated MLH1 methylation is linked to relapse and mortality, though a study with a larger sample size would be essential for statistically significant results.

**Abstract:**

*Background*: In cancer care, the MLH1 gene is crucial for DNA mismatch repair (MMR), serving as a vital tumor suppressor. Evaluating MLH1 protein expression status, followed by analysis of MLH1 promoter methylation, has become a key diagnostic and prognostic approach. Our study investigates the complex link between MLH1 methylation and prognosis in endometrial adenocarcinoma (EA) patients. *Methodology*: MLH1 methylation status was accessed by a Pyrosequencing (PSQ) assay. Qualitative positivity for methylation was established if it exceeded the 11% cut-off; as well, a quantitative methylation analysis was conducted to establish correlations with clinicopathological data, relapse-free survival, and disease-free survival. *Results:* Our study revealed that 33.3% of patients without MLH1 methylation experienced relapses, surpassing the 23.3% in patients with methylation. Furthermore, 16.7% of patients without methylation succumbed to death, with a slightly higher rate of 17.6% in methylated patients. Qualitative comparisons highlighted that the mean methylation rate in patients experiencing relapse was 35.8%, whereas in those without relapse, it was 42.2%. This pattern persisted in disease-specific survival (DSS), where deceased patients exhibited a higher mean methylation level of 49.1% compared to living patients with 38.8%. *Conclusions*: Our findings emphasize the efficacy of PSQ for evaluating MLH1 methylation. While unmethylation appears to be associated with a higher relapse rate, the survival rate does not seem to be influenced by methylation. Quantitative percentages suggest that elevated MLH1 methylation is linked to relapse and mortality, though a study with a larger sample size would be essential for statistically significant results.

## 1. Introduction

Endometrial cancer (EC), the most common form of uterine cancer, presents a significant health challenge for women worldwide. In 2020, approximately 417,000 new cases of EC were identified, with the highest occurrence rates noted in developed nations. The typical age of diagnosis is around 60 years, and the incidence tends to rise with advancing age. Moreover, EC exhibits a higher prevalence among white women in comparison to black women, while Asian/Pacific Islander women show the lowest incidence rates [1].

Obesity stands as a significant risk factor for EC, increasing the risk by two to three times in obese women compared to those with a normal weight [2]. Other risk factors include unopposed estrogen exposure, nulliparity, early menopause, and polycystic ovary syndrome (PCOS) [3]. Early detection and treatment of EC have led to substantial improvements in survival rates, with a five-year survival rate exceeding 80% for localized disease [1,4].

The International Federation of Gynecology and Obstetrics (FIGO) has devised a staging system to categorize EC, which was recently updated in 2023. This system is based on the degree of tumor spread and is essential for guiding treatment choices and predicting patient outcomes [5]. The FIGO staging system for EC serves as a standardized framework for classifying the extent of the disease and guiding treatment decisions. This system is crucial for enhancing patient care and improving overall outcomes [6].

In stage I, standard treatment often involves surgery and close monitoring to catch any disease relapse. For young women who wish to preserve their fertility, progestin therapy might be used instead of immediate surgery. Stage II treatment typically involves a radical hysterectomy, removal of both ovaries and fallopian tubes (bilateral salpingo-oophorectomy), and dissection or sampling of pelvic and para-aortic lymph nodes. After recovery from surgery, patients usually undergo radiotherapy, which may include vaginal brachytherapy and external pelvic radiation, or radiotherapy might be administered before surgery. Lymph nodes removed during surgery are examined for cancer cells. Stage III EC extended beyond the uterus. In stage IIIA, treatment after surgery often includes chemotherapy, radiation, or both, targeting the pelvis or abdomen, along with vaginal brachytherapy. In stage IIIB, the post-surgery regimen may consist of chemotherapy and/or radiation. For stage IIIC, the treatment plan includes surgery followed by chemotherapy and/or radiation. In most stage IV, extensive metastasis makes surgery impractical. However, a hysterectomy and removal of both ovaries and fallopian tubes may still be performed to control severe bleeding, with radiotherapy used as an additional treatment. Hormonal therapy might be applied for widespread cancer, but it is generally ineffective against high-grade cancers that lack progesterone and estrogen receptors [7,8].

In EC treatment, chemotherapy often involves drug combinations to enhance efficacy. A common regimen includes paclitaxel, doxorubicin, and either cisplatin or carboplatin. Paclitaxel works by inhibiting cell division, thus preventing cancer cells from multiplying. Doxorubicin interferes with DNA replication, leading to cell death. Cisplatin and carboplatin are platinum-based drugs that cause DNA damage and induce apoptosis. Administering these drugs in combination allows for a multifaceted attack on the cancer cells, improving treatment outcomes compared to single-drug therapies. In advanced cases, targeted therapies such as tyrosine kinase inhibitors and immunotherapies like PD-1/PD-L1 inhibitors may be employed. These treatments can specifically target cancer cells or enhance the immune system’s ability to recognize and destroy them, offering additional options when standard chemotherapy is insufficient [9,10].

In addition to accurate histopathological diagnosis, the emergence of molecular biology has provided valuable insights into improving cancer patient prognosis. Based on a more comprehensive genomic analysis, TCGA identified four prognostic subtypes of EC: ultra-mutated DNA polymerase epsilon (POLE), hypermutated microsatellite instability (MSI), low-copy number, and high-copy number. Patients with ultra-mutated POLE genes exhibit impaired DNA proofreading during replication, resulting in a hypermutator phenotype. This subtype typically benefits from immunotherapy due to the immunogenic nature of neoantigens produced by the increased number of somatic mutations. Affected patients are usually younger and present with early-stage tumors that are high-grade and exhibit significant lymphocytic infiltration. Despite the higher grade of these tumors, they generally have a favorable prognosis. Patients with MSI-high tumors also respond well to immunotherapy, particularly checkpoint inhibitors, due to their high mutational load. A common approach combines immunotherapy with the current standard systemic therapy: chemotherapy with carboplatin and paclitaxel, followed by maintenance immunotherapy. Despite these treatments, MSI-high tumors have an intermediate prognosis and are limited to effective systemic therapies. Low-copy number types are often early-stage cases with a favorable prognosis and a lower risk of recurrence. This molecular subtype is typically lower-grade with positive estrogen and progesterone receptors, making it generally sensitive to hormonal therapy due to its association with estrogen exposure. In contrast, the high copy number subtype is characterized by high genomic instability and frequent TP53 mutations, leading to a worse prognosis and a poorer response to standard therapies [11,12,13,14].

Integrating clinical and pathological characteristics to inform treatment decisions, such as using concurrent radiochemotherapy, chemotherapy, inhibitor therapy, endocrine therapy, and immunotherapy, could enhance the management of endometrial cancer and offer patients more effective treatment options [15].

**Figure 1:**
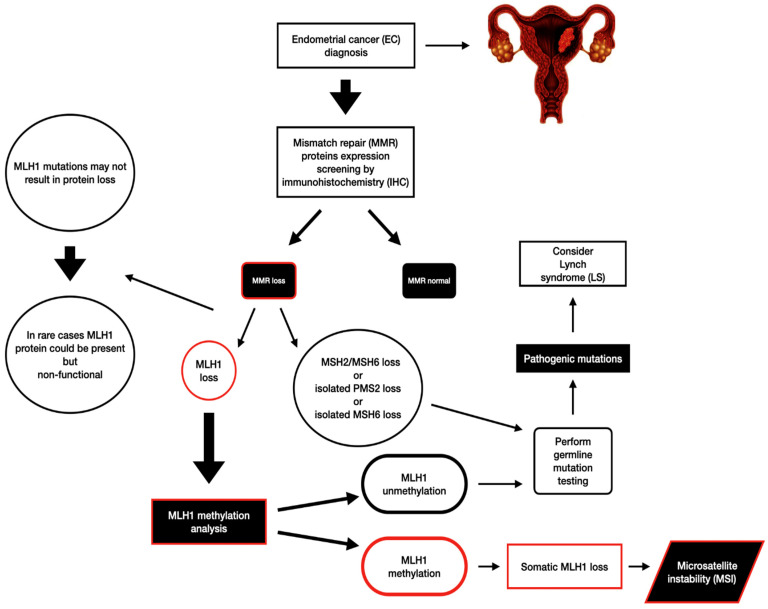
MLH1 screening in endometrial cancer (EC). A cascade of molecular screening is initiated to guide treatment decisions, assess prognosis, and identify associated hereditary syndromes. The initial step involves analyzing mismatch repair (MMR) protein expression through immunohistochemistry (IHC). MSH2/MSH6, isolated PMS2 loss, or isolated MSH6 loss cases require germline mutation testing. Specifically, in instances of MLH1 loss, germline mutation testing is performed to detect potential pathogenic mutations, particularly those indicative of Lynch syndrome (LS). Additionally, when MLH1 loss is observed, methylation analysis is necessary. Unmethylated MLH1 prompts further germline mutation testing, while methylated MLH1 suggests somatic loss, indicative of microsatellite instability (MSI).

The MLH1 gene, a crucial tumor-suppressor gene, plays a central role in DNA mismatch repair (MMR). MMR is an essential process that ensures the faithful replication of DNA during cell division, preventing the accumulation of errors that can contribute to cancer development [11]. Mutations in MLH1 or any other MMR gene are correlated with Lynch syndrome (LS) development. This inherited condition significantly increases the risk of various cancers, including EC. LS is a hereditary condition that can generate a greater likelihood of specific cancers, although it mainly affects the colon, endometrium, ovaries, and urinary tract [16]. The risk of EC in this scenario can climb as high as 60% throughout a woman’s lifespan [17]. Diagnosing LS involves pinpointing specific mutations in the DNA MMR genes mentioned earlier (MLH1, PMS2, MSH2, and MSH6) [18]. A common screening method involves examining tissue samples from hysterectomies for abnormalities in protein expression using IHC. If loss of MLH1 and PMS2 protein expression is detected, further analysis checks for methylation on the MLH1 gene promoter. This additional step helps differentiate sporadic cases from inherited ones [19,20] (Figure 1).

Patients whose tumors show MLH1 unmethylation, those with specific protein expression patterns indicating missing pieces in the MSH2/MSH6 partnership, or those lacking just PMS2 or MSH6 protein, require more comprehensive genetic testing [19,21]. This deeper dive aims to identify the exact mutation responsible for their increased cancer risk. An important aspect of this molecular screening is its role in guiding therapeutic decisions for these cancer patients. Additionally, it facilitates the initiation of regular check-ups for other family members if germline mutations are identified [22,23,24].

Pyrosequencing (PSQ), a sequencing-by-synthesis approach, utilizes real-time nucleotide incorporation and tracks enzymatic pyrophosphate conversion into a proportional light signal for quantitative analysis. Quantitative measurements are critical for DNA methylation analysis in various developmental and pathological contexts. PSQ-based DNA methylation analysis combines a straightforward reaction protocol with reproducible and precise measurements of methylation levels at multiple CpG sites within proximity, providing high quantitative resolution [25].

This retrospective observational study (non-experimental) involved the careful analysis of medical records of patients diagnosed with endometrial adenocarcinoma (EA) at the Hospital Clânico Universitário de Santiago de Compostela in Santiago de Compostela, Spain, during the period from January 2017 to March 2023. The period in which the PSQ began to be used in our hospital center aimed to compare the results of MLH1 methylation by the PSQ with clinicopathological factors and the evolution of these patients. We are the only group responsible for receiving all genetic analyses regarding the MLH1 gene in cancer in the Galician population; therefore, our aim is also to provide a database in the literature that can serve for future ethnogeographical associations of the methylation of this gene since this is a factor that must always be taken into consideration when it comes to cancer research.

## 2. Results

### 2.1. Sample Description

Out of the 40 patients diagnosed with EA, the average age at diagnosis was 67.9 years, ranging from 39 to 92 years. At the time of diagnosis, 77.5% (31) of the patients were over 60 years old. In terms of tumor stage according to FIGO staging classification, 52.5% (21) of patients were classified as stage I, 25% (10) as stage II, 20% (8) as stage III, and 2.5% (1) as stage IV. More specifically, considering the subgroups from the last FIGO staging classification update: 37.5 (15) were classified as stage IA; 15% (6) as IB; 15% (6) as IIA; 5% (2) as IIB; 5% (2) as IIC; 10% (4) as IIIB1; 5% (2) as IIIC1i; 2.5% (1) as IIIC1ii; 2.5% (1) as IIIC2ii; and 2.5% (1) as stage IV. As of December 2023, 25% (10) of patients had relapsed, and 17.5% (7) of the patients diagnosed between February 2017 and April 2023 were dead (Table 1).

### 2.2. IHC

**Figure 2:**
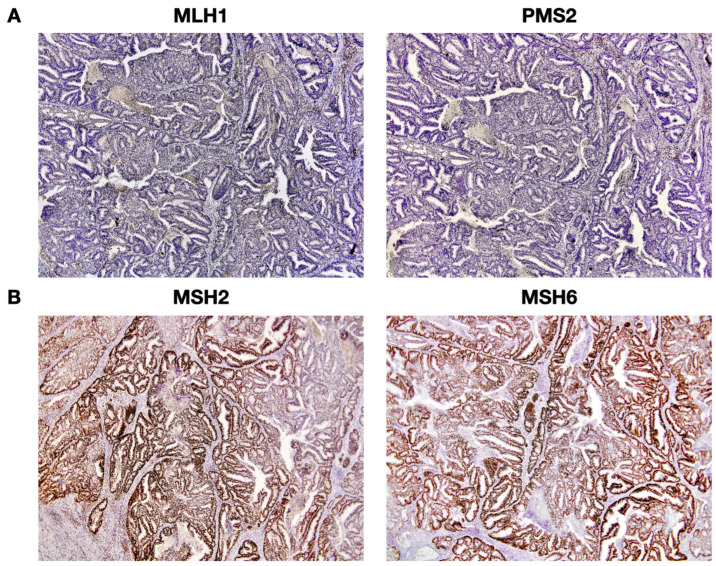
Representative protein expression analysis in immunohistochemistry (IHC). (**A**) The absence of diffuse cytoplasmatic staining is indicative of loss of MLH1 and PMS2 protein expression; (**B**) Positivity of MSH2 and MSH6 expression is indicated by significant cytoplasmatic staining. The antibodies used were anti-MLH1, anti-PMS2, anti-MSH2, and anti-MSH6 (Ready-to-use; Agilent, DAKO Omnis, Santa Clara, CA, USA); 20× magnification.

Of the 28 patients whose p53 protein status was checked, 92.9% (26) had a p53 wild-type status. When analyzing the density of estrogen receptor-positive cells, of the 28 patients, 100% were positive, with an average of 76.2% of positive cells, while the density of cells positive to progesterone receptors, of the 28 patients, 92.9% of patients were positive, with an average of 68.7% of positive cells. Of the 16 patients whose vimentin expression was analyzed, 75% (12) were positive for protein expression. As one of the inclusion criteria in our study was the loss of MLH1 expression, the 40 patients presented this characteristic, as well as the loss of expression of its heterodimer, the PMS2 protein. Conversely, of all 40 patients, all showed positive expression of MSH2 and its heterodimer, the MSH6 protein. Figure 2 demonstrates a representative IHC standard for the 4 MMR proteins mentioned in the same patient included in our study.

### 2.3. Aberrant Methylation of the MLH1 Gene in Patients with EA

**Figure 3:**
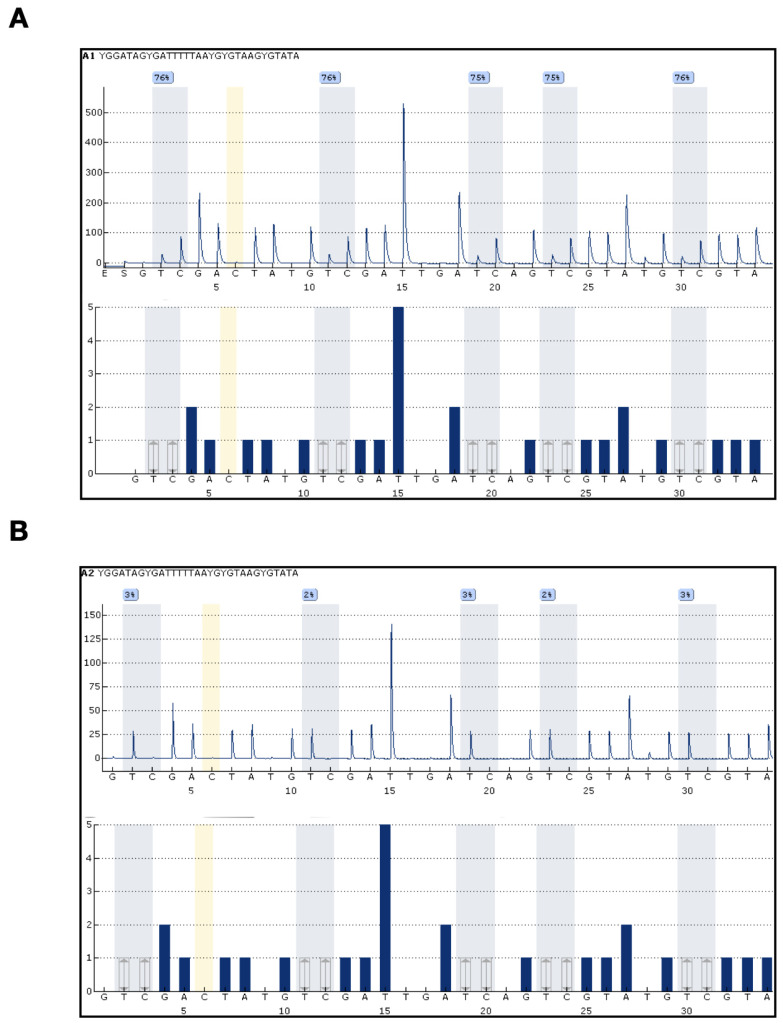
Representative pyrosequencing (PSQ) to determine MLH1 methylation status. (**A**) Exemplifies a hypermethylated case with average methylation levels across five CpG islands (island 1 = 76%; island 2 = 76%; island 3 = 75%; island 4 = 75%; island 5 = 76%), resulting in a representative average of 75.6%; (**B**) Depicts an unmethylated case with average methylation levels across four CpG islands (island 1 = 3%; island 2 = 2%; island 3 = 3%; island 4 = 2%; island 5 = 3%), yielding a representative average of 2.6%. The positivity cut-off is set at 11%.

The percentage of patients who presented with MLH1 methylation was 90%. Considering all 40 patients, the average methylation was 40.6%, ranging from 1.4% to 83.4%. A clear association of hypermethylation with loss of MLH1 protein expression was observed in patients diagnosed with EA, as can be observed through our results. The widespread silencing of DNA MMR genes, evident by aberrant methylation, could be a key driver of MSI in many patients. This impaired repair mechanism allows mutations to accumulate in repetitive DNA sequences, potentially contributing to the development of EA. Notably, our data highlights the MLH1 gene as a critical player in this process, with its abnormal methylation mirroring the high prevalence of MSI in EA patients. Figure 3 demonstrates a representative PSQ to determine MLH1 methylation status, with (A) a hypermethylated case and (B) an unmethylated case.

### 2.4. Association of Clinicopathological Factors with MLH1 Methylation Status

Within this cohort, age at diagnosis demonstrated a slight association with methylation levels, with patients aged 60 or above exhibiting a mean methylation rate of 44.7%, considerably higher compared to 26.4% in younger individuals. Similarly, disease stage correlated with methylation, displaying a stepwise increase: Stage 1 (36%), Stage 2 (47%), Stage 3 (55.7%), and Stage 4 (67.4%) (Table 1). The consistently elevated median methylation levels observed across clinicopathological factors suggest a lack of influence and a widespread presence of methylation in a significant proportion of our sample; however, we observed an association between increased methylation percentage and patients over 60 years old and disease stage. 

### 2.5. Association of Molecular Factors with MLH1 Methylation Status

Patients who presented with the p53 abnormal protein had an average methylation rate of 67.7%, a number considerably higher than the 40.8% of p53 wild-type patients. Patients who showed negative vimentin expression had an average methylation of 24.8%, while patients with positive expression had a higher average of 35.6%. Patients with more than 50% density of estrogen receptor-positive cells had an average methylation of 51.5%, a much higher percentage when considering those below 50%, which was 14.8%. Patients with more than 50% density of progesterone receptor-positive cells had an average methylation of 50.3%, a slightly higher percentage when considering those below 50%, which was 42.5%. We observe some associations between certain molecular characteristics and methylation percentages; however, the divergences between the number of patients in each group, as this is an observational study, should be considered.

### 2.6. Prognostic and Outcome Implications of MLH1 Methylation in EA

#### 2.6.1. Qualitative Methylation Status

When comparing qualitative methylation status, 33.3% of unmethylated patients experienced relapses, which is higher than the 23.3% relapse rate observed in methylated patients. Regarding disease-specific survival (DSS), in our results, 16.7% of unmethylated patients died, while for methylated patients, this rate was slightly higher, at 17.6%. These results demonstrate a disagreement between methylated patients and their association with relapses and survival, where MLH1 methylation presented a lower chance of relapse but a greater chance of survival.

#### 2.6.2. Quantitative Methylation Status

**Figure 4:**
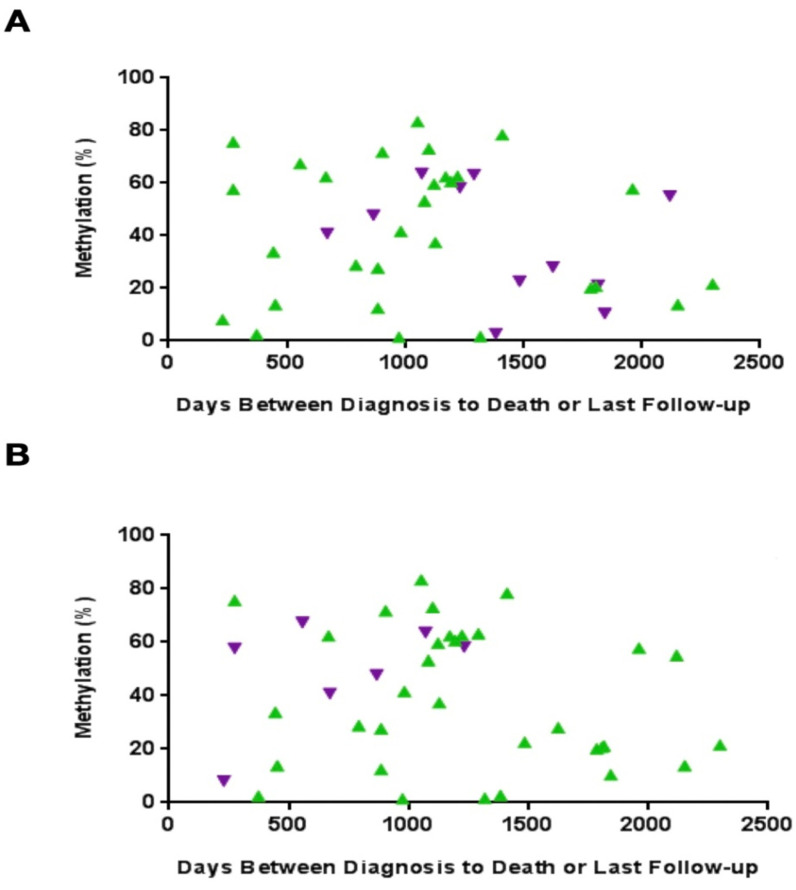
Comparative analysis of qualitative methylation status in patients reveals distinct outcomes. Despite group-level trends indicating lower relapse and higher survival chances with MLH1 methylation, attempts to establish a statistical model linking individual methylation values to patient outcomes lack significance (*p*-values of 0.33 and 0.64), attributed to the limited number of relapses and disease-related deaths within our sample size. The purple points represent (**A**) relapsed and (**B**) dead patients; and the green represents (**A**) non-relapsed and (**B**) alive patients.

In a quantitative comparison, the mean methylation rate was 35.8% in patients who experienced relapse, compared to 42.2% in those who did not relapse. DSS reflected this trend, with deceased patients exhibiting a higher mean methylation level (49.1%) compared to living patients (38.8%). Increased MLH1 methylation is associated with relapse and mortality, suggesting a quantitative link with prognostic factors. However, while attempting to construct a model relating individual methylation values to patient relapses and survival, our results found that despite the observed trends with group averages, the association lacked statistical significance with p-values of 0.33 and 0.64, justified by the limited number of patients that experienced relapses and disease-related deaths within our sample size (Figure 4).

## 3. Discussion

Methylation of the MLH1 promoter is frequently linked to sporadic tumors exhibiting MSI, such as colorectal and EC. This methylation correlates with the loss of MLH1 protein expression, detectable through IHC. Therefore, in cancer types characterized by high-level MSI or loss of MLH1 staining via IHC, the absence of MLH1 promoter methylation implies a higher probability of germline mutation in a DNA MMR protein [18,25,26].

While the association between MLH1 methylation and LS is generally considered rare, it is important to note that the absence of MLH1 methylation is not conclusive for diagnosis. This is because approximately 1% of LS cases result from constitutional methylation of the MLH1 promoter. Hence, a comprehensive evaluation involving the patient’s medical history and prior IHC and genetic tests is essential for an accurate diagnosis [27,28].

The analysis of MLH1 methylation is limited in its ability to exclude the existence of epigenetic modifications beyond the evaluated promoter region, and it does not identify point mutations, insertions, deletions, or inversions. This test is designed for application in tumors exhibiting high-level MSI and/or experiencing loss of MLH1 staining via IHC [19,29].

Our methodology included only patients exhibiting loss of MLH1 protein expression via IHC as an inclusion criterion, as this subgroup is considered more valid for methylation analysis in assessing tumor MSI. Bruegl et al. analyzed without this inclusion criterion in their study, revealing results indicating the detection of loss of MLH1 nuclear expression by IHC in only 16% of endometrial tumors. Within this subset, 74.1% demonstrated MLH1 promoter methylation and were classified as sporadic EC [30].

Metcalf et al. observed MLH1 methylation in 94% of tumors with MLH1 loss by IHC, as well as with studies by Buchanan et al. and Toboni et al., wherein MLH1 methylation was identified in 89% and 89.2% of cases, respectively, showing sole loss of MLH1 expression by IHC [31,32,33]. Our findings align with these results exhibiting loss of MLH1 expression via IHC, 90% displayed methylation of the MLH1 gene. In contrast, Jisup Kim et al. reported a lower percentage, with 69.2% of cases exhibiting a methylated MLH1 promoter, differing from the notably higher percentages mentioned earlier [34].

The mean methylation level in our study was 40.6%, revealing aberrant hypermethylation predominantly in patients experiencing IHC loss of the MLH1 protein. Notably, the average methylation increased to 44.7% in patients aged 60 and above, while those below this age exhibited a lower average of 26.4%. This observation underscores the significance of age as an important factor in the context of somatic mutations [35]. For instance, Turashvili et al. reported that in their study, 95% of EA patients demonstrated somatic mutations through MLH1 methylation, particularly in individuals with advanced ages [36].

A significant limitation emerged when comparing our methylation percentage results across different molecular conditions due to the lack of outcome homogeneity. Most patients exhibited p53 wild-type, a high density of cells positive for estrogen and progesterone receptors, and vimentin positivity. In the molecular profiles analyzed, these changes in methylation percentages arouse greater interest in future, more robust associations since, as our research is a retrospective observational study, there are inherent differences in sample sizes between each group that may affect the results. For example, only two patients presented p53 abnormal. It is also important to clarify that when we consider patients with a loss of MLH1 protein expression as an inclusion factor, the expression results of other proteins in these patients become more homogeneous.

Recent research in EA has identified several promising biomarkers that could revolutionize diagnosis, prognosis, and treatment. Among these, the L1 cell adhesion molecule (L1CAM) has emerged as a critical prognostic marker, associated with aggressive tumor behavior and poor outcomes. Additionally, abnormalities in the TP53 tumor suppressor gene and mutations in the PTEN gene have been linked to the development and progression of this cancer. The discovery of MSI and POLE exonuclease domain mutations provides further insight into tumor behavior and potential therapeutic targets. Advances in genomic and proteomic technologies are also uncovering a range of novel biomarkers, including specific microRNAs and long non-coding RNAs, which may offer new avenues for early detection and personalized treatment strategies in endometrial adenocarcinoma [37].

In our results, a qualitative assessment of the MLH1 methylation status using an 11% cut-off revealed a diminished likelihood of relapse but an enhanced probability of survival. The survival outcomes, albeit suggestive, may lack conclusiveness given the predominant occurrence of stage I disease in most patients, which is inherently associated with heightened survival rates. Nevertheless, the association of unmethylated status with an increased risk of relapses aligns coherently with the potential linkage of these patients to LS [38].

In the context of a qualitative methylation analysis, patients who did not experience relapse exhibited a slightly higher average methylation rate, mirroring a similar trend in DSS. Deceased patients displayed a higher mean methylation level compared to their living counterparts. These findings align with the observations of Loukovaara et al., whose study reported poorer DSS in MLH1-methylated cases compared to those proficient in MMR [39].

Toboni et al. corroborated our findings by showing that EC patients with MLH1 methylation experience significantly poorer survival outcomes and distinct immunotherapy responses compared to those with MLH1-mutated tumors [33]. In concordance with these findings, a recent study by Kaneko et al. classified EC into three categories based on MMR status: MMR-proficient, suspected-LS, and methylated-EC. The study demonstrated that methylated cases have a poorer prognosis compared to other ECs with MMR deficiencies. Consequently, patients with met-EC should be prioritized as primary candidates for anti-PD-1 antibody treatment [40].

Conversely, Shikama et al. presented contrasting results, indicating that the prognostic significance of MLH1 methylation in EC did not correlate with overall survival. This was consistent across sporadic cases and those with unmethylated MMR, characterized by intact expression of all MMR proteins or loss of MLH1 expression but with methylation of the MLH1 promoter [41].

Beyond simply assessing the correlation of methylation with clinicopathological and prognostic factors, our findings represent a notable advancement in incorporating PSQ-based analysis of MLH1 gene methylation. Although numerous other methodologies have been investigated to improve the reliability of such analyses, challenges regarding inconsistencies persist [42,43,44]. There is a pressing need to identify a more effective technique and establish more definitive parameters of positivity for unquestionable prognostic associations. PSQ emerges as a robust and viable method to achieve this goal, notwithstanding its current classification as an expensive technique, and our study represents a pioneering endeavor to access MLH1 methylation in EA patients using PSQ.

Nonetheless, according to Buchanan et al., their study emphasizes that the comprehensive pre-selection of patients based on routine clinical, histopathological, and molecular characteristics renders the MLH1 methylation test more specific. Furthermore, the MLH1 methylation test is considered a more cost-effective alternative to BRAF V600E mutation testing, as this mutation occurs infrequently in EC and is therefore not considered a practical alternative predictor for negative germline MMR mutation status [32].

The principal limitation in our study stems from the significant heterogeneity observed between the groups of methylated and unmethylated patients. However, this diversity can be readily explained by our inclusion criteria, which specifically selected patients exhibiting loss of MLH1 protein expression by IHC. Consequently, the predominance of methylated patients is justified.

Similarly, substantial heterogeneity is noted in the number of patients experiencing relapse versus those who did not, as well as between alive and deceased patients. This variability can be attributed to the same heterogeneity in clinicopathological factors, such as staging and age, which directly influence the patient’s prognosis.

Given that our study is a retrospective analytical observational study, it is imperative to acknowledge that the analyses were not tailored specifically for research objectives. This imposes constraints on our findings, particularly concerning sample size. It is crucial to underscore the significance of our study, recognizing that the analysis pertains to a distinct population and acknowledging the substantial influence of ethnographic factors on genetic variables. Despite these limitations, our previous findings indicate promise for comprehensive exploration of the topic in future experimental studies. Such endeavors could enhance comprehension and utilization of specific factors known to enhance prognosis in patients with endometrial carcinoma (EC).

## 4. Materials and Methods

### 4.1. Patient Selection

This retrospective observational study included a patient group selected from the Santiago de Compostela University Clinical Hospital. The period from 2017 to 2023 was stipulated because it was the period in which the technique began to be used in our hospital center. The study inclusion and exclusion criteria were primarily centered on retrospectively analyzing MLH1 methylation results in patients with EA, specifically those who underwent pyrosequencing analysis. Subsequently, these data were compared with clinicopathological and prognostic information. Clinical and pathological records were sourced from the Galician Health Service database. Certain comparisons involving histopathological characteristics were omitted due to insufficient patient numbers in each group.

The study’s inclusion criteria were: Diagnosis of EA; loss of MLH1 protein expression; MLH1 gene methylation analysis performed using PSQ; availability of follow-up data up to either death or the last follow-up in December 2023. Patients were excluded from the study if: absence of IHC to detect MLH1/PMS2 and MSH2/MSH6 expression; MLH1 gene methylation analysis using PSQ was not conducted; insufficient follow-up data were available.

### 4.2. Ethical Approval

The study protocol was reviewed and approved by the Santiago-Lugo Research Ethics Committee (CEI-SL), with registration code 2023/157. All procedures performed, as they were studies involving human participants, were in accordance with the Declaration of Helsinki.

### 4.3. IHC

The IHC procedure began with the dewaxing and rehydration of 5 µm paraffin-embedded tissue sections using a suitable dewaxing solution and graded alcohols, ensuring complete removal of the paraffin wax. Antigen retrieval was then performed using EnVision™ FLEX target retrieval solution (Agilent, DAKO, Santa Clara, CA, USA) at pH 10 for 20 min at 95 °C. Slides were cooled and treated with EnVision™ FLEX peroxidase-blocking reagent solution (Agilent, DAKO, USA) for 5 min. Sections were incubated with anti-MLH1, anti-PMS2, anti-MSH2, and anti-MSH6 (Ready-to-use; Agilent, DAKO Omnis, Santa Clara, CA, USA) primary antibodies in EnVision™ FLEX antibody diluent and incubated with the diluted antibody at room temperature for 20 min. Complete immunostaining was performed using the EnVision™ FLEX + Mouse LINKER/HRP (Agilent, DAKO, Santa Clara, CA, USA) technique following the manufacturer’s instructions. The staining evaluation of the histopathological slides was always carried out by an expert anatomopathologist, and positivity was considered whenever the nuclei staining was diffused throughout the entire area of the tumor.

### 4.4. Analysis of MLH1 Methylation

#### 4.4.1. PSQ

To precisely evaluate the methylation status of the MLH1 gene in the samples, a PSQ assay was employed. The procedure began with total DNA extraction from the paraffin-embedded samples, effectively isolating the genetic material for subsequent analysis. Next, bisulfite treatment was performed using the Epitect Fast DNA Bisulfite Kit (Qiagen^®^, Hilden, Germany) according to the manufacturer’s instructions. This step involved meticulously dissecting a single 5 µm tissue section from the biopsy specimens. Using a NanoDrop 2000 (Thermo Fisher Scientific, Waltham, MA, USA), the sample concentrations were accurately measured, yielding 260/280 ratios within the acceptable range of 1.7 to 2 for all samples. The modified DNA, now containing both methylated and unmethylated cytosine bases, was extracted for further analysis. PCR amplification was conducted at a concentration of no more than 100 ng/μL using the Pyromark PCR kit 200 (Qiagen^®^). PSQ was performed employing the Pyromark Q24 CPG MLH1 (4 × 24) (Qiagen^®^) probe. All PCR reactions were performed on an Agilent Technologies Surecycler 8800 system, while pyrosequencing was carried out on a Pyromark Q24 and Pyromark Q24 workstation (Qiagen^®^). The Pyromark Q24 2.0.7 software was utilized to analyze the pyrosequencing data, providing a clear determination of the methylation status of the MLH1 gene in each sample [45].

#### 4.4.2. Quantification of Methylation

To accurately establish the methylation status of each sample, the average methylation levels of the five CpG islands were individually calculated. Based on the guidelines provided by the MLH1 Pyro kit (Qiagen^®^, Hilden, Germany), a methylation level of 11% or higher was considered indicative of a positive methylation status (methylated). For ambiguous cases where the methylation status remained unclear, a more in-depth examination of the corresponding CpG island and Pyrogram was conducted to refine the assessment.

### 4.5. Data Extraction

The qualitative variables evaluated in this study included: FIGO staging classification; date of diagnosis; date of death (if applicable); survival status; follow-up duration; tumor recurrence; MLH1, PMS2, MSH2, MSH6, vimentin, p53 proteins expressions status; density of cells positive to estrogen and progesterone receptors; MLH1 methylation percentages; and a range of methylation values.

### 4.6. Statistical Analysis

This study complied with the Reporting Recommendations for Tumor Marker Prognostic Studies (REMARK) guidelines [36,46,47]. Statistical analysis was performed using an unpaired Student’s *t*-test in GraphPad Prism version 6 (GraphPad Software Inc., La Jolla, CA, USA), with *p*-values < 0.05 considered statistically significant [48].

## 5. Conclusions

Our findings underscore an elevated prevalence of EA patients exhibiting a methylated MLH1 gene concurrent with loss of MLH1 protein expression in IHC. Moreover, they highlight PSQ as a viable and reliable method for assessing MLH1 methylation status. Regarding prognostic factors, a qualitative evaluation of MLH1 methylation suggests an association between unmethylation and increased relapse rates, potentially linked to germline mutations and LS. Conversely, DSS rates do not appear to be significantly influenced by methylation status, although unmethylated patients exhibit a marginally higher survival rate. Quantitative analysis indicates that elevated MLH1 methylation levels are correlated with higher rates of relapse and mortality; however, achieving statistical significance would necessitate a study with a larger sample size.

## Figures and Tables

**Table 1:** Qualitative variables in comparison with levels of methylation.

Qualitative Variables		Number of Patients	Average MLH1 Methylation
Age	<60 years old	22.5% (9)	26.4%
	≥60 years old	77.5% (31)	44.7%
FIGO Staging	I	52.5% (21)	36%
	II	25% (10)	47%
	III	20% (8)	55.7%
	IV	2.5% (1)	67.5%
Relapses	Non-relapsed	75% (30)	42.2%
	Relapsed	25% (10)	35.8%
Exitus	Alive	82.5% (33)	38.8%
	Dead	17.5% (7)	49.1%

## Data Availability

The raw data supporting the conclusions of this article will be made available by the authors on request.

## References

[1] Makker V., MacKay H., Ray-Coquard I., Levine D.A., Westin S.N., Aoki D., Oaknin A. (2021). Endometrial Cancer. Nat. Rev. Dis. Primers.

[2] Wang X., Glubb D.M., O’Mara T.A. (2023). Dietary Factors and Endometrial Cancer Risk: A Mendelian Randomization Study. Nutrients.

[3] Raglan O., Kalliala I., Markozannes G., Cividini S., Gunter M.J., Nautiyal J., Gabra H., Paraskevaidis E., Martin-Hirsch P., Tsilidis K.K. (2019). Risk factors for endometrial cancer: An umbrella review of the literature. Int. J. Cancer.

[4] Cicinelli E., Ballini A., Marinaccio M., Poliseno A., Coscia M.F., Monno R., De Vito D. (2012). Microbiological findings in endometrial specimen: Our experience. Arch. Gynecol. Obstet..

[5] Berek J.S., Matias-Guiu X., Creutzberg C., Fotopoulou C., Gaffney D., Kehoe S., Lindemann K., Mutch D., Concin N., Wilailak S. (2023). FIGO staging of endometrial cancer: 2023. Int. J. Gynaecol. Obstet. Off. Organ Int. Fed. Gynaecol. Obstet..

[6] Leitao M.M. (2024). 2023 changes to FIGO endometrial cancer staging: Counterpoint. Gynecol. Oncol..

[7] Crosbie E.J., Kitson S.J., McAlpine J.N., Mukhopadhyay A., Powell M.E., Singh N. (2022). Endometrial cancer. Lancet.

[8] Oaknin A., Bosse T.J., Creutzberg C.L., Giornelli G., Harter P., Joly F., Lorusso D., Marth C., Makker V., Mirza M.R. (2022). Endometrial cancer: ESMO Clinical Practice Guideline for diagnosis, treatment and follow-up. Ann. Oncol. Off. J. Eur. Soc. Med. Oncol..

[9] Tubridy E.A., Taunk N.K., Ko E.M. (2024). Treatment of node-positive endometrial cancer: Chemotherapy, radiation, immunotherapy, and targeted therapy. Curr. Treat. Options Oncol..

[10] Pados G., Zouzoulas D., Tsolakidis D. (2024). Recent management of endometrial cancer: A narrative review of the literature. Front. Med..

[11] Peterson L.M., Kipp B.R., Halling K.C., Kerr S.E., Smith D.I., Distad T.J., Clayton A.C., Medeiros F. (2012). Molecular characterization of endometrial cancer: A correlative study assessing microsatellite instability, MLH1 hypermethylation, DNA mismatch repair protein expression, and PTEN, PIK3CA, KRAS, and BRAF mutation analysis. Int. J. Gynecol. Pathol. Off. J. Int. Soc. Gynecol. Pathol..

[12] Urick M.E., Bell D.W. (2019). Clinical actionability of molecular targets in endometrial cancer. Nat. Rev. Cancer.

[13] Soberanis Pina P., Lheureux S. (2024). Novel Molecular Targets in Endometrial Cancer: Mechanisms and Perspectives for Therapy. Biol. Targets Ther..

[14] Ogunmuyiwa J., Williams V. (2024). Emerging Advances in Endometrial Cancer: Integration of Molecular Classification into Staging for Enhanced Prognostic Accuracy and Implications for Racial Disparities. Cancers.

[15] Yang Y., Wu S.F., Bao W. (2024). Molecular subtypes of endometrial cancer: Implications for adjuvant treatment strategies. Int. J. Gynecol. Obstet..

[16] Cerretelli G., Ager A., Arends M.J., Frayling I.M. (2020). Molecular pathology of Lynch syndrome. J. Pathol..

[17] Zhao S., Chen L., Zang Y., Liu W., Liu S., Teng F., Xue F., Wang Y. (2022). Endometrial cancer in Lynch syndrome. Int. J. Cancer.

[18] Salem M.E., Bodor J.N., Puccini A., Xiu J., Goldberg R.M., Grothey A., Korn W.M., Shields A.F., Worrilow W.M., Kim E.S. (2020). Relationship between MLH1, PMS2, MSH2 and MSH6 gene-specific alterations and tumor mutational burden in 1057 microsatellite instability-high solid tumors. Int. J. Cancer.

[19] Beiner M.E., Rosen B., Fyles A., Harley I., Pal T., Siminovitch K., Zhang S., Sun P., Narod S.A. (2006). Endometrial cancer risk is associated with variants of the mismatch repair genes MLH1 and MSH2. Cancer Epidemiol. Biomark. Prev..

[20] Ørbo A., Nilsen M.N., Arnes M.S., Pettersen I., Larsen K. (2003). Loss of expression of MLH1, MSH2, MSH6, and PTEN related to endometrial cancer in 68 patients with endometrial hyperplasia. Int. J. Gynecol. Pathol. Off. J. Int. Soc. Gynecol. Pathol..

[21] Stelloo E., Jansen A.M.L., Osse E.M., Nout R.A., Creutzberg C.L., Ruano D., Church D.N., Morreau H., Smit V.T.H.B.M., van Wezel T. (2017). Practical guidance for mismatch repair-deficiency testing in endometrial cancer. Ann. Oncol. Off. J. Eur. Soc. Med. Oncol..

[22] Favier A., Varinot J., Uzan C., Duval A., Brocheriou I., Canlorbe G. (2022). The Role of Immunohistochemistry Markers in Endometrial Cancer with Mismatch Repair Deficiency: A Systematic Review. Cancers.

[23] Lacey J.V., Yang H., Gaudet M.M., Dunning A., Lissowska J., Sherman M.E., Peplonska B., Brinton L.A., Healey C.S., Ahmed S. (2011). Endometrial cancer and genetic variation in PTEN, PIK3CA, AKT1, MLH1, and MSH2 within a population-based case-control study. Gynecol. Oncol..

[24] Terzic M., Aimagambetova G., Kunz J., Bapayeva G., Aitbayeva B., Terzic S., Laganà A.S. (2021). Molecular Basis of Endometriosis and Endometrial Cancer: Current Knowledge and Future Perspectives. Int. J. Mol. Sci..

[25] Bruchim I., Capasso I., Polonsky A., Meisel S., Salutari V., Werner H., Lorusso D., Scambia G., Fanfani F. (2024). New therapeutic targets for endometrial cancer: A glimpse into the preclinical sphere. Expert Opin. Ther. Targets.

[26] Bruno V., Logoteta A., Chiofalo B., Mancini E., Betti M., Fabrizi L., Piccione E., Vizza E. (2024). It is time to implement molecular classification in endometrial cancer. Arch. Gynecol. Obstet..

[27] Carnevali I.W., Cini G., Libera L., Sahnane N., Facchi S., Viel A., Sessa F., Tibiletti M.G. (2023). MLH1 Promoter Methylation Could Be the Second Hit in Lynch Syndrome Carcinogenesis. Genes.

[28] Kahn R.M., Gordhandas S., Maddy B.P., Baltich Nelson B., Askin G., Christos P.J., Caputo T.A., Chapman-Davis E., Holcomb K., Frey M.K. (2019). Universal endometrial cancer tumor typing: How much has immunohistochemistry, microsatellite instability, and MLH1 methylation improved the diagnosis of Lynch syndrome across the population?. Cancer.

[29] Russell H., Kedzierska K., Buchanan D.D., Thomas R., Tham E., Mints M., Keränen A., Giles G.G., Southey M.C., Milne R.L. (2020). The MLH1 polymorphism rs1800734 and risk of endometrial cancer with microsatellite instability. Clin. Epigenetics.

[30] Bruegl A., Djordjevic B., Urbauer D., Westin S., Soliman P., Lu K., Luthra R., Broaddus R. (2014). Utility of MLH1 methylation analysis in the clinical evaluation of Lynch Syndrome in women with endometrial cancer. Curr. Pharm. Des..

[31] Metcalf A.M., Spurdle A.B. (2014). Endometrial tumour BRAF mutations and MLH1 promoter methylation as predictors of germline mismatch repair gene mutation status: A literature review. Fam. Cancer.

[32] Buchanan D.D., Tan Y.Y., Walsh M.D., Clendenning M., Metcalf A.M., Ferguson K., Arnold S.T., Thompson B.A., Lose F.A., Parsons M.T. (2014). Tumor mismatch repair immunohistochemistry and DNA MLH1 methylation testing of patients with endometrial cancer diagnosed at age younger than 60 years optimizes triage for population-level germline mismatch repair gene mutation testing. J. Clin. Oncol. Off. J. Am. Soc. Clin. Oncol..

[33] Toboni M.D., Wu S., Farrell A., Xiu J., Ribeiro J.R., Oberley M.J., Arend R., Erickson B.K., Herzog T.J., Thaker P.H. (2023). Differential outcomes and immune checkpoint inhibitor response among endometrial cancer patients with MLH1 hypermethylation versus MLH1 "Lynch-like" mismatch repair gene mutation. Gynecol. Oncol..

[34] Kim J., Kong J.K., Yang W., Cho H., Chay D.B., Lee B.H., Cho S.J., Hong S., Kim J.H. (2018). DNA Mismatch Repair Protein Immunohistochemistry and MLH1 Promotor Methylation Testing for Practical Molecular Classification and the Prediction of Prognosis in Endometrial Cancer. Cancers.

[35] Hitchins M.P., Alvarez R., Zhou L., Aguirre F., Dámaso E., Pineda M., Capella G., Wong J.J.L., Yuan X., Ryan S.R. (2023). MLH1-methylated endometrial cancer under 60 years of age as the “sentinel” cancer in female carriers of high-risk constitutional MLH1 epimutation. Gynecol. Oncol..

[36] Turashvili G., Colgan T., McLachlin M., Lin H., Gharbharan R. (2022). Lynch Syndrome Screening of Women with Endometrial Cancer: Feasibility and Outcomes in a Community Program. J. Obstet. Gynaecol. Can..

[37] Iavarone I., Molitierno R., Fumiento P., Vastarella M.G., Napolitano S., Vietri M.T., De Franciscis P., Ronsini C. (2024). MicroRNA Expression in Endometrial Cancer: Current Knowledge and Therapeutic Implications. Medicina.

[38] Jiang W., Gao T., Tao X., Zhu M., Yao L., Feng W. (2019). Endometrioid endometrial cancer “recurring” as high-grade serous adeno-carcinoma in the inguinal lymph nodes in a patient with germline MLH1 mutated Lynch syndrome: Consequence or coincidence?. Hered. Cancer Clin. Pract..

[39] Kaneko E., Sato N., Sugawara T., Noto A., Takahashi K., Makino K., Terada Y. (2021). MLH1 promoter hypermethylation predicts poorer prognosis in mismatch repair deficiency endometrial carcinomas. J. Gynecol. Oncol..

[40] Loukovaara M., Pasanen A., Bützow R. (2021). Mismatch repair protein and MLH1 methylation status as predictors of response to adjuvant therapy in endometrial cancer. Cancer Med..

[41] Shikama A., Minaguchi T., Matsumoto K., Akiyama-Abe A., Nakamura Y., Michikami H., Nakao S., Sakurai M., Ochi H., Onuki M. (2016). Clinicopathologic implications of DNA mismatch repair status in endometrial carcinomas. Gynecol. Oncol..

[42] Galbraith K., Snuderl M. (2022). DNA methylation as a diagnostic tool. Acta Neuropathol. Commun..

[43] Plotkin A., Olkhov-Mitsel E., Nofech-Mozes S. (2023). MLH1 Methylation Testing as an Integral Component of Universal Endometrial Cancer Screening-A Critical Appraisal. Cancers.

[44] Higashimoto K., Hara S., Soejima H. (2023). DNA Methylation Analysis Using Bisulfite Pyrosequencing. Methods Mol. Biol..

[45] e Silva F.F.V., Di Domenico M., Caponio V.C.A., Pérez-Sayáns M., Camolesi G.C.V., Rojo-Álvarez L.I., Ballini A., García-García A., Padín-Iruegas M.E., Suaréz-Peñaranda J.M. (2024). Pyrosequencing Analysis of O-6-Methylguanine-DNA Methyltransferase Methylation at Different Cut-Offs of Positivity Associated with Treatment Response and Disease-Specific Survival in Isocitrate Dehydrogenase-Wildtype Grade 4 Glioblastoma. Int. J. Mol. Sci..

[46] Sauerbrei W., Taube S.E., McShane L.M., Cavenagh M.M., Altman D.G. (2018). Reporting Recommendations for Tumor Marker Prognostic Studies (REMARK): An abridged explanation and elaboration. J. Natl. Cancer Inst..

[47] Altman D.G., McShane L.M., Sauerbrei W., Taube S.E. (2012). Reporting Recommendations for Tumor Marker Prognostic Studies (REMARK): Explanation and elaboration. PLoS Med..

[48] Mitteer D.R., Greer B.D. (2022). Using GraphPad Prism’s Heat Maps for Efficient, Fine-Grained Analyses of Single-Case Data. Behav. Anal. Pract..

